# Nanotechnology in the Diagnosis and Treatment of Antibiotic-Resistant Infections

**DOI:** 10.3390/antibiotics13020121

**Published:** 2024-01-25

**Authors:** Petros Ioannou, Stella Baliou, George Samonis

**Affiliations:** 1 School of Medicine, University of Crete, 71003 Heraklion, Greece; 2 First Department of Medical Oncology, Metropolitan Hospital of Neon Faliron, 18547 Athens, Greece

**Keywords:** nanotechnology, antimicrobial resistance, Gram-negative, Gram-positive, multi-drug resistant, extensively-drug resistant, pan-drug resistant

## Abstract

The development of antimicrobial resistance (AMR), along with the relative reduction in the production of new antimicrobials, significantly limits the therapeutic options in infectious diseases. Thus, novel treatments, especially in the current era, where AMR is increasing, are urgently needed. There are several ongoing studies on non-classical therapies for infectious diseases, such as bacteriophages, antimicrobial peptides, and nanotechnology, among others. Nanomaterials involve materials on the nanoscale that could be used in the diagnosis, treatment, and prevention of infectious diseases. This review provides an overview of the applications of nanotechnology in the diagnosis and treatment of infectious diseases from a clinician’s perspective, with a focus on pathogens with AMR. Applications of nanomaterials in diagnosis, by taking advantage of their electrochemical, optic, magnetic, and fluorescent properties, are described. Moreover, the potential of metallic or organic nanoparticles (NPs) in the treatment of infections is also addressed. Finally, the potential use of NPs in the development of safe and efficient vaccines is also reviewed. Further studies are needed to prove the safety and efficacy of NPs that would facilitate their approval by regulatory authorities for clinical use.

## 1. Introduction

Infectious diseases are an important cause of mortality worldwide [1]. During the last decades, there has been a reduction in morbidity and mortality of these communicable diseases in higher-income countries; however, infectious diseases are still a cause for significant morbidity, even in these countries [2,3,4]. Importantly, antimicrobial resistance (AMR) is a global problem that poses a significant threat to millions of people [5,6]. For example, 4.95 million deaths were estimated to be associated with bacterial AMR in 2019, and it is predicted that this number will greatly increase [5]. Unfortunately, the pharmaceutical pipeline has not met the growing need for antimicrobials to combat AMR [7]. Thus, academia and the pharmaceutical industry have also tried to identify methods that involve treatments other than antimicrobial use to fight resistant pathogens that very few, if any, antimicrobials are potent to kill. To that end, non-antibiotic treatments may include simple nutrients or amino-acids, such as D-mannose, antimicrobial peptides, bacteriophages, or even more technically elegant methods, such as nanobiotics [8,9,10,11,12].

Nanoscience involves the use of materials on the scale of nanometers in an attempt to develop technology (nanotechnology) that could have several applications in electronics or medicine [13]. More specifically, several applications of nanotechnology in medicine have been developed in the last decades, such as the development of liposomes [14,15], polymer-drug conjugates [16], DNA-drug complexes [17], polymer nanocapsules [18,19], antibody-drug conjugates [20], albumin-drug conjugates [21], polymer-protein conjugates [22], antimicrobial silver nanoparticles [23], and gold nanoparticles for rheumatologic disorders [24,25]. Nanotechnology has numerous applications in medicine, such as in drug delivery, where several nanostructures can be used for the direct delivery of a specific drug to a specific biological target with controlled drug release and reduced toxicity, and in in vivo imaging with nanoparticles used as imaging contrast agents, such as in computed tomography and magnetic resonance imaging [26]. Other applications of nanotechnology in medicine include in vitro diagnosis, tissue regeneration and engineering, as well as its use with implantable or wearable devices for diagnosis or treatment [26].

This study aimed to provide an overview of the current medical applications of nanotechnology in infectious diseases and, more specifically, against pathogens with significant AMR. In particular, applications of nanotechnology in the diagnosis and treatment of infectious diseases are discussed, along with specific representative examples of the different technologies that have been developed so far.

## 2. Search Methodology

This narrative review aims to present the basic principles of the applications of nanotechnology in the diagnosis and treatment of infectious diseases with a focus on pathogens with AMR, and it also aims to provide representative examples of the different technologies that have been developed so far. Importantly, this manuscript was written from a clinician’s perspective; thus, its main focus is to inform the reader of the spectrum of the applications of nanotechnology in infectious diseases by providing the necessary emphasis on technical information and, more importantly, underlining the current and future potential of nanotechnology.

For this review, a search of the PubMed/Medline database up until 18 January 2024 for eligible articles was conducted, using the search terms ‘nanotechnology AND infectious diseases’. Relevant reviews and original studies providing information on the topic were retrieved, evaluated, and added to the evidence synthesis in a liberal, non-systematic way. Two investigators (PI and SB) performed the process of screening, extracting, and synthesizing the evidence. The references of the included articles were searched for the identification of other relevant articles.

## 3. Principles of Nanotechnology

The basic functional unit of a material used in nanotechnology is a nanoparticle (NP). NPs are composed of metal, metal oxides, carbon, or organic matter and have higher surface charge, stability, strength, reactivity, sensitivity, surface area, and absorption compared to other older materials [27]. Their size is in the nanoscale, most commonly between 1 and 100 nm [28]. They have unique magnetic, electric, optical, or other properties in the nanoscale that allow them to function, and they have been recently placed at the center of many research studies because of their great potential to produce materials, with uses in several fields, such as medicine and many others, like industry [13,28,29].

Even though this field of research may sound innovative and quite recent, the principles of nanotechnology have been used for a long time, as in the case of the reinforcement of ceramic matrices with natural asbestos nanofibers approximately 4500 years ago or in the case of lead sulfide (PbS) NPs with a size of approximately 5 nm that were chemically synthesized in the same period for use in hair dye [28]. Ever since, principles of nanotechnology have been used through metallic NPs, Cu NPs, Ag NPs, Au NPs, or SiO_2_ NPs for decorative purposes, antibacterial use, and rubber reinforcement, among other uses [28].

Some common types of NPs include carbon nanotubes, dendrimers, liposomes, metallic NPs, micelles, and quantum dots. Carbon nanotubes are cylindrical molecules consisting of folded sheets of carbon atoms that may be single-walled, multi-walled, or that may be formed by several nanotubes that are concentrically interlinked [13,30]. They can cross cell membranes in both an endocytosis-independent and an endocytosis-dependent manner [31,32]. They can serve as efficient carriers for drugs due to their high external surface [13]. Moreover, they have optical, electronic, and mechanical properties that allow them to be used as biological sensors and imaging contrast agents [32,33,34]. They can be used in photoacoustic imaging, drug delivery, phototherapy, gene delivery, photoluminescence imaging, thermal therapy, gas storage, diagnostics, and elsewhere [35]. Some of their disadvantages are their relatively low solubility in water, the variable pharmacokinetics that depend on their physicochemical characteristics, and the potential for toxicity, more likely to the lungs [36]. Dendrimers are highly ordered macromolecules that have branching repeating functional units and usually have symmetry and a spherical three-dimensional morphology [13,37,38,39]. These molecules can have cationic, neutral, or anionic terminals, and they can encapsulate therapeutic molecules in their interior or at their surface and, thus, facilitate their delivery due to their bioavailability. They can be conjugated to peptides or saccharides to enhance their antimicrobial activity [40]. More specifically, they can be used as conjugates to drug molecules, fluorescent trackers, antibodies, enzymes, and targeting ligands that can be released either actively or passively to their target; they could also be used as drug carriers through the encapsulation of the drug or as gene carriers through complexation with the target gene [41]. Some of their limitations include the difficulty in their production as well as their toxicity, which depends on their net charge as well as on their size, with dendrimers of different sizes being also eliminated from different routes [41]. For example, smaller dendrimers have renal elimination, while larger ones rely on hepatic elimination [41]. Liposomes are spherical particles with a size as low as 30 nm that are composed of lipid bilayers. They are typically used to enhance the delivery of drugs by incorporating them in their hydrophilic center, if the drugs are hydrophilic, or into their hydrophobic membrane, if the drugs are hydrophobic [13]. Their characteristics can also be modified with peptides, antibodies, or polymers, allowing for the formation of macromolecular drugs [38]. Several liposomes are currently in clinical use, mostly in oncology but also in the treatment and prevention of infectious diseases [42]. Some of their limitations include the relatively high cost of production, their short half-life, their low solubility, and the possibility of leakage of the encapsulated drug [43]. Metallic NPs mainly refer to gold and iron NPs. Iron oxide NPs are composed of a magnetic core 4–5 nm in size and hydrophilic polymers such as dextran [30,32,34]. Gold NPs consist of a gold atom core in the center and negative reactive groups in the periphery that can be modified with the addition of surface moieties, such as ligands, to provide active molecular targeting [13,30,32,34]. They have the potential to carry proteins, nucleic acids, drugs, cell-penetrating agents, imaging agents, or specific targeting moieties, such as monoclonal antibodies [44]. Their biosynthesis can be mediated by microorganisms and plants, which leads to a lower production cost [45]. Several clinical studies are currently ongoing and are evaluating the activity and safety of metallic NPs in the treatment of cancer and infectious diseases [44]. Some of their limitations may include issues regarding biocompatibility and their relatively weak optical signal [46,47]. Micelles are surfactants of lipids with amphiphilic properties due to their polarized heads. They have the ability to assemble spherical vesicles in a spontaneous manner in aqueous conditions, having a hydrophobic inner core and a hydrophilic outer layer due to the hydrophilic head of their amphiphilic molecules [48]. They have several applications, mainly due to their ability to solubilize hydrophobic drugs, thus increasing their bioavailability [13]. For this reason, they are used as imaging agents, therapeutics, and drug delivery agents [13,49]. More specifically, there are several clinical trials with micelles being evaluated in the treatment of various cancers, such as breast, lung, bladder, and pancreatic cancer [50]. One of their limitations includes their relatively limited loading capacity [51,52,53]. Quantum dots are nanocrystals with fluorescent semiconductor properties with the potential for use in cellular imaging and drug delivery [30,54,55]. They have a shell structure consisting of elements from groups II-VI or III-V of the periodic table and have optical properties and sizes that allow them to be used in medical imaging [13,55]. Several clinical trials with quantum dots in the detection and diagnosis of cancer, acute myocardial infarction, and type one diabetes mellitus are ongoing [56]. Moreover, their potential in the detection of cells expressing specific target receptors is also being evaluated [56]. Some of their limitations include pharmaceutical issues regarding their relative instability that could lead to aggregation or chemical redox alterations [56]. Furthermore, the requirements for their production could make their mass production difficult. Moreover, the possibility for adverse reactions and their non-specificity for interaction with all host cells are factors that may limit their use. Table 1 shows a comparison of the characteristics, advantages, and disadvantages of the different NPs.

**Figure 1:**
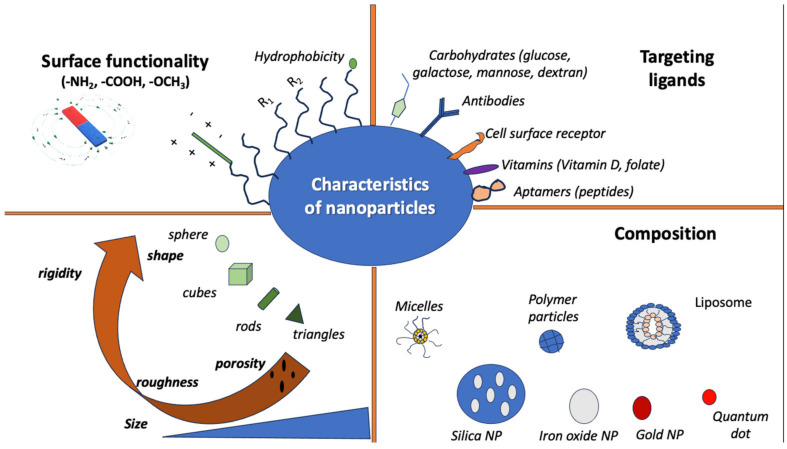
Characteristics of nanoparticles in terms of shape, composition, targeting ligands, and surface function.

Additionally, the use and properties of NPs depend on factors like shape and size. More specifically, the function of NPs often depends on their surface area, which provides a relatively high area-to-volume index, given their very small size [28]. Some of their possible properties include the following: mechanical, thermal, optical, electronic, and magnetic properties. Mechanical properties include hardness, friction, interfacial adhesion, and elastic modulus, among others, and they are primarily used in surface engineering, nanofabrication, painting materials, and nanomanufacturing [28,64,65]. The optical and electronic properties of NPs are interconnected, and they are mostly used in light-emitting diodes (LEDs), sensors, biophotonics, batteries, and semiconductors [28,66,67]. The magnetic properties of NPs seem apparent in NPs of 10–20 nm in size that have uneven electron distribution [28]. NPs with magnetic properties are used in catalysts, data storage, water and soil purification, magnetic resonance imaging (MRI), and elsewhere [65,68,69]. Figure 1 shows the characteristics of different nanoparticles.

A new field, nanomedicine, has been developed, encompassing all of these nanotechnology applications in medicine. Several applications have emerged, such as cancer, cardiovascular disease, diabetes, neurodegenerative diseases, and infectious diseases. In infectious diseases, nanomedicine has applications in diagnosis as well as in therapy [13,38,70,71,72,73]. More specifically, nanomedicine in the field of infectious diseases may aid in the diagnosis and treatment of bacterial, viral, parasitic, or mycobacterial disease [74,75,76,77,78,79]. The following sections of this review specifically address the applications of nanotechnology in diagnosing and treating infectious diseases.

## 4. Nanotechnology in the Diagnosis of Infectious Diseases

### 4.1. Basic Principles in the Diagnosis of Infectious Diseases

Due to the large area-to-volume ratio and the reactivity of the molecules at the nanomaterials’ surface, these nanomaterials have great potential in diagnosing infectious diseases. The use of nanomaterials as diagnostic tools has been attributed to their ability to bind infectious agents and occupy mechanical, optical, electronic, and magnetic signals [80]. They also provide the ability to perform point-of-care diagnostic tests, identify resistance determinants, and provide multiplexing capacity, thus being of utmost importance in diagnosis [81,82,83]. Until now, many nanobiosensing assays have been used in the diagnosis of infectious diseases in humans, but their efficacy and safety remain to be elucidated [80,84,85]. Different principles occupied by nanosensors include colorimetric, electrochemical, fluorescent, surface-enhanced Raman spectroscopy (SERS), and others [86].

### 4.2. Nanosensors Occupying Colorimetric Properties

Colorimetric biosensors create visible signals to the naked eye and have long been used in diagnosing infectious diseases [86]. One of their primary disadvantages is their relatively low sensitivity, which may limit their broad use. Nanomaterials with optical properties may provide a higher sensitivity in such assays in diagnosing infectious diseases [86]. Nanotechnology offers many different substrates for use as nanosensors. For example, gold NPs (AuNPs) are commonly used in that direction due to their unique surface plasmon resonance (SPR) phenomenon that gives them strong absorption in visible light [86]. Thus, many sensitive colorimetric assays have been developed occupying AuNPs, such as assays for the detection of pathogen nucleic acid in paper-based biosensors combined with loop-mediated amplification (LAMP) [86,87]. In this case, the biosensor functions in nucleic acid extraction, amplification, and detection. After the amplification, amplicons labeled with biotin bind to AuNPs that are detection probes. Then, due to the biotin–streptavidin interaction, this complex is immobilized at the test zone and emits a visible signal that is read as a positive test [86]. Other techniques also take advantage of the optical abilities of AuNPs, such as in the case of enzyme-linked immunosorbent assays (ELISA) that are used for the detection of biomarkers with increased sensitivity [88,89,90]. Other nanomaterials, such as AgNPs, are occupied in colorimetric applications of nanotechnology for diagnosing infectious diseases [91,92,93]. Another application of NPs has to do with their potential to be used as enzymes. These NPs, called nanozymes, have similar catalytic activity but may have increased stability compared to enzymes [86,94]. One such example involves the use of copper-based metal-organic framework (Cu-MOF) NPs with enzymatic activity similar to peroxidase that is used for the detection of *Staphylococcus aureus* [95].

### 4.3. Nanosensors Occupying Electrochemical Properties

The electrochemical properties of some NPs are important for the detection of pathogens [86,96]. The principle of the method relies on the production of measurable electrochemical signals upon pathogen detection, such as impedance, current, or potential. Hence, nanomaterials based on carbon, such as graphene or carbon nanotubes, are used for the production of biosensors due to their high conductivity [86]. For example, the detection of uropathogenic *Escherichia coli* is possible using a multiwall carbon nanotube-chitosan composite-based electrochemical immunosensor, while this technique has also been used for the detection of genetic material, like in the case of human papillomavirus DNA [97,98]. An example of a biosensor occupying the measurement of potential is the potentiometric graphene-based aptasensor that was developed for detecting *S. aureus* in a concentration of even a single cfu/mL level in just one or two minutes [99].

### 4.4. Nanosensors Occupying Fluorescent Properties

Fluorescent biosensors are of great value in pathogen detection assays due to their high sensitivity, even though these assays may be limited because of the relatively low stability of fluorophores and their tendency to bleach [86]. Nanosensors with fluorescent properties have been evaluated for diagnosing infectious diseases, with various examples available. Hence, quantum dots may be valuable tools in that direction due to their narrow emission peak, their high photostability, and tunable emission wavelength [100,101]. Likewise, CdSe@ZnS quantum dots are used in a lateral flow assay for pathogen detection [101]. More specifically, the conjugates of antibodies with quantum dots are immobilized at the test and the control lines. Graphene oxide is added; thus the quantum dots are quenched if pathogens are absent. However, in the presence of pathogens, the distance between quantum dots and the graphene oxide is increased, allowing fluorescence detection from the quantum dots [101]. Other examples of NPs used as biosensors due to their fluorescent properties include silver nanoclusters, along with graphene oxide for the detection of pathogen DNA, carbon dots that, similar to quantum dots, have the photoluminescent emission properties that can be used for bioimaging and bioanalysis, such as, for example, in the case of Gram-positive bacterial detection [102,103,104].

### 4.5. Nanosensors Occupying SERS Properties

SERS is a sensitive method that uses optical detection [86,105]. Its principle relies on the enhancement of Raman scattering by nanomolecules, such as plasmonic-magnetic nanotubes or by molecules adsorbed on rough metal surfaces [106]. This method allows for the enhancement of scattering intensity up to 10^14^ times, thus making this a very sensitive method for diagnosis even at a single-molecule level [86,107,108]. This method has been used for viral detection. An SERS-based lateral flow assay has been used for the detection of HIV-1 genetic material or the detection of the DNA of Kaposi sarcoma-associated herpesvirus [109,110].

### 4.6. Nanosensors in Point-of-Care Testing

Point-of-care testing has the advantage of providing diagnostic abilities to people who do not have either the expertise or the high technical capacities nearby, thus allowing the use of sophisticated technology in the field where the diagnostic abilities of these tests are mostly needed [86]. Such examples of point-of-care testing include the use of magnetic NPs. For example, magnetic NP-DNA complexes and conjugates of DNA-invertase are used for the detection of hepatitis B virus (HBV) DNA [111]. More specifically, the DNA fragments of HBV are captured by the magnetic NPs labeled with a DNA fragment partially complementary to the target DNA. Then, the DNA hybridizes with the DNA-invertase conjugate. After separation of the sandwich complex and incubation with sucrose, glucose is produced, and this can be measured with a glucose meter for the quantification of the DNA fragment [111]. Other point-of-care tests have been developed for microorganism detection, such as for severe acute respiratory syndrome coronavirus 2019 (SARS-CoV-2) [112]. Nanotechnology can also occupy newer scientific and technological methods, such as CRISP-Cas technology or even smartphone-based technology [113,114,115]. Table 2 shows some examples of nanosensors occupying different principles that have been evaluated in diagnosing infectious diseases.

### 4.7. Future Perspectives in Nanotechnology and the Diagnosis of Infectious Diseases

The current era provides huge potential for the advancement of diagnostics in infectious diseases. The implementation of the abovementioned principles of nanotechnology in the diagnosis of infectious diseases provides a great opportunity for diagnosing infections with high sensitivity and specificity, even with point-of-care tests. Optimization of the diagnostic techniques, overcoming technical barriers to increase sensitivity and specificity, large-scale production of the tests, and reduction of cost should be the next steps toward the increased implementation of this technology in the diagnosis of infectious diseases. Mass production and reduction of cost are of particular significance since many of the tests that are either available or under development could be of great use in countries with limited resources but with a high burden of disease, such as in the case of HIV infection and AIDS in Africa [122].

Moreover, the introduction of artificial intelligence in medicine could also aid in the diagnosis of infectious diseases. Combining nanotechnology applications in diagnosis with artificial intelligence could also lead to more potent diagnostic tools through increasing automation in diagnosis, reducing human error, increasing speed in providing diagnostic results, and increasing diagnostic accuracy [122,123,124].

## 5. Nanotechnology in the Treatment of Infectious Diseases

### 5.1. The Need for Non-Antibiotic Interventions in the Treatment of Infectious Diseases

Even though antimicrobials have changed the face of human history by allowing the effective treatment of many infectious diseases and also enabling surgeons to conduct major surgical managements with significant reduction of surgical site infections, antimicrobial resistance developed rapidly after the initiation of antimicrobial use, thus making some of the previously mentioned treatments unavailable [125]. The development of extensively-drug-resistant and pan-drug-resistant pathogens has posed severe therapeutic limitations in the everyday clinical practice of infectious diseases [126,127]. Given the imbalance in the relatively slow production of new antimicrobials, from the pharmaceutical pipeline and the rapid development of AMR, new non-classical therapeutic options could be of great use in the fight against pathogens with significant AMR [128]. Several therapeutic strategies have been employed in this direction involving natural and chemical products, probiotics and prebiotics, antimicrobial peptides, bacteriophages, predatory bacteria, immunotherapy, vaccines, and other methods [10,11,129,130,131,132,133]. Nanotechnology may offer valuable tools in this fight against pathogens harboring significant AMR.

### 5.2. Basic Principles of Nanotechnology in the Treatment of Infectious Diseases

Due to their small size and specific electrical, magnetic, and binding properties, the NPs could be used in the fight against infectious diseases, even against resistant pathogens. Their properties and size could allow them to easily cross bacterial membranes and target specific biosynthetic and enzymatic pathways [134,135]. This is in contrast to classic antimicrobials that may not enter in adequate concentrations inside the target cells due to the rarity of pores and the transport mechanisms needed to enter the cells, as well as due to mechanisms employed by target cells aimed towards antimicrobial drug efflux outside the cell or its enzymatic inhibition [134,136]. NPs may act through various mechanisms against pathogens. More specifically, they may have inherently antibacterial activity or act as delivery vehicles for antibiotics that may be carried on or inside them. In these cases, they are called nanobiotics or nanoantibiotics [134]. Inorganic NPs may have inherent antibiotic activity and present many different mechanisms of antibacterial activity. These are called nanobacteriocides, while NPs acting as NP-based delivery systems transferring old antibiotics are called nanocarriers [134].

### 5.3. Nanotechnology and Enhanced Drug Delivery in the Treatment of Infectious Diseases

Nanocarriers may be used to treat infectious diseases due to their ability to transfer antibiotics to the target, thus altering the pharmacological properties of antibiotics by increasing their absorption or increasing their delivery to the tissues and the target microbial cells [134,137,138,139]. One typical example is encochleated amphotericin B, a novel formulation of a well-known antifungal drug [138,139]. Encochleated amphotericin B is a novel nanoparticle-based formulation that encapsulates and delivers amphotericin B in the cells. It does so by protecting amphotericin B in the interior of an anhydrous crystal of calcium and phospholipids [139]. This formulation completely alters the pharmacokinetics of the drug, allowing, for the first time now, the oral administration of this drug that had for decades been administered only intravenously [139]. However, this drug has not yet received FDA approval for clinical use.

Liposomes are the most commonly used NPs for enhancing drug delivery for topical and systemic use. They are appropriate for both hydrophobic and hydrophilic molecules [134]. Liposomes have multiple advantages that make them ideal candidates for drug delivery. They are non-toxic and biodegradable since they comprise material resembling human cell membranes. Moreover, they can be modified in multiple ways, including size, lipid composition, and net surface charge, allowing modification of their half-life, their pharmacokinetics, and the way they carry the drug to be delivered [134]. Finally, they have the ability to fuse with membranes, either those of the human cells or the ones of the pathogens, and this allows for the delivery of the antibiotic inside the pathogen or even inside the human cells in the case of infection by an intracellular pathogen [140,141]. An example of liposomes used as carrier molecules in the therapeutics of infection includes the delivery of antiretrovirals, such as zidovudine or indinavir in the case of infection by HIV that leads to more efficient delivery of the drug to the lymphoid tissues compared to administration without liposomes [142,143,144].

Dendrimers can also be used as carriers of drugs in the treatment of infectious diseases. Their hyper-branched structure with the central core and the three-dimensional branches can increase the solubility of hydrophobic drugs, thus increasing their efficacy [145,146]. For example, the delivery of efavirenz with dendrimers has been studied, leading to the enhanced delivery of the drug to cells of the immune system [147].

### 5.4. Nanoparticles as Antimicrobial Drugs

Metallic NPs can have toxic effects on microorganisms since they may induce the production of reactive oxygen species under specific circumstances, such as under ultraviolet light [148]. Metallic NPs with Au, Ag, Ti, Cu, Zn, Fe, Ti, or metal oxides can have significant antimicrobial activity against bacteria, viruses, or fungi [145,149,150,151,152,153,154,155].

For example, AgNPs act as antimicrobials based on four mechanisms: binding to the cell of the microorganisms, destabilization of its cell membrane and induction of changes in its permeability, generation of reactive oxygen species and free radicals, leading to cell toxicity of the microorganisms, and induction of alterations in signal transduction within the pathogen [156,157]. As a first step towards their antimicrobial activity, AgNPs bind to the pathogen’s membrane, mainly driven by their net charge. More specifically, the AgNPs with a more positive net charge have an increased antimicrobial effect due to their higher ability to bind to the pathogen’s membrane, even though a high concentration of AgNPs can also lead to a potent antimicrobial effect through the saturation of the microbial surface [156,158]. After binding to the microorganism’s cell membrane, small AgNPs can penetrate the membrane, while larger AgNPs may remain on the microorganism’s membrane. In both cases, the NPs release Ag+ ions, destabilizing the microorganism’s membrane and causing leakage of the contents of the microbial cell. This membrane destabilization may allow even large AgNPs to enter the pathogen, thus allowing them to act intracellularly as well [159]. After entering the target microorganism, AgNPs, and the Ag+ ions that are released, may interact with various structures of the microorganism, such as proteins, DNA, and lipids, blocking several critical biological functions. They can produce free radicals, reactive oxygen species, and oxidative stress that interact with carboxyl, thiol, and phosphate groups of proteins, thus affecting their activity and blocking microbial growth [156,160]. Finally, AgNPs were shown to have better penetration in bacterial biofilms in a pH-dependent manner. This leads to the persistence of AgNPs in the biofilms for a long time, the disruption of bacterial biofilm formation, and enhanced antibacterial activity [161].

The antimicrobial activity of AgNPs against wild strains of *Salmonella* was examined in a study by Losasso et al. [159]. AgNPs of spherical and sporadically regular polygonal shape with a median diameter of 6 nm and 18 nm appeared at the electron microscope. The addition of AgNPs effectively reduced counts in a dose-dependent manner, with doses of 200 mg/L being the most effective [159]. In the same study, the authors also described the mechanism of resistance of *Salmonella* to AgNPs. Expression of the SilB gene, which has been implicated in bacterial resistance to silver and copper, was confirmed and was further identified to be positioned on the plasmidic portion of the bacterial genetic material [159].

Similarly, CuONPs have potent antimicrobial effects by first binding on the microbial cell wall through electrostatic interactions and then by the delivery of Cu^2+^ ions that lead to an intracellular increase of reactive oxygen species as well as to increased permeability of the microbial wall membrane, leading to the leakage of intracellular material [162]. To this end, the antibacterial activity of CuONPs has been shown against many bacteria, including *V. cholera*, *P. aeruginosa*, *E. coli*, *S. aureus*, and *E. faecalis* [156,163,164,165].

More specifically, in a relatively old study, Sabbatini et al. established polymer-based NPs loaded with CuNPs [155]. Copper was embedded in three different polymers, namely, poly-(vinyl chloride) (PVC), polyvinylmethyl ketone (PVMK), and polyvinylidenefluoride (PVDF), leading to the formation of CuNPs of 80–530 nm in size. The in vitro kinetics of copper release were examined, and the CuNPs were shown to have potent antimicrobial activity against *Saccharomyces cerevisiae*, *Escherichia coli*, *Staphylococcus aureus*, and *Listeria monocytogenes* [155].

In a more recent study, Lv et al. used *Shewanella loihica* PV-4 as a means for the production of CuNPs that were purified, characterized with electron microscopy, and found to have a size of 6–20 nm [165]. In vitro application of 100 µg/mL of the CuNPs could inhibit 86% of *E. coli*. The mechanism of action of CuNPs was studied, and the bacterial cells had become amorphous and wizened after a 12 h incubation with the CuNPs. The bacterial membrane had accumulated CuNPs as well as lack of its continuity [165]. Deposits of CuNPs were also seen inside the bacterial cells and could be associated with bacterial damage [165].

AuNPs have antibacterial activity against Gram-negative and Gram-positive bacteria, such as *P. aeruginosa*, *E. coli*, *K. pneumoniae*, *S. aureus*, and *E. faecalis* [156]. Even though AuNPs also bind to the bacterial surface based on electrostatic interactions, depending on their net charge, they are thought to have minimal inherent antibacterial activity; thus, their mechanism of action mostly relies on the induction of changes in the bacterial membrane potential. Moreover, they may also reduce the adenosine triphosphatase (ATPase) activity, leading to a reduction in the bacterial ATP, and inhibit protein translation by inhibiting tRNA binding on the ribosomes [156,166]. Gold ion release and reactivity leading to the production of reactive oxygen species are considered to contribute to a much lower extent in the activity of AuNPs [156].

In a study by Nagalingam et al., AuNPs were synthesized using leaf extracts of *Alternanthera bettzickiana*. Using scanning electron microscopy, the AuNPs were identified as spherical, and they had a size of 80–120 nm. The zeta potential was found to be −41.4. The AuNPs had adequate antimicrobial activity against *S. typhi*, *P. aeruginosa*, *E. aerogenes*, *S. aureus*, *B. subtilis*, and *M. luteus* [167].

In another study by Arockiya Aarthi Rajathi et al., biosynthesis of AuNPs by the brown alga *Stoechospermum marginatum* was performed [168]. Scanning electron microscopy revealed that polydispersed NPs were formed and had a size that was within the range of 40–85 nm. Transmission electron microscopy revealed that the AuNPs had variable shapes and were mostly spherical, even though triangular and hexagonal NPs were also seen. The AuNPs had adequate antibacterial activity when tested against *P. aeruginosa*, *K. oxytoca*, *E. faecalis*, *K. pneumoniae*, *V. cholerae*, *E. coli*, *S. typhi*, *S. paratyphii*, *V. parahaemolyticus*, and *P. vulgaris* [168].

ZnONPs exhibit antimicrobial activity that spans Gram-positive and Gram-negative bacteria, such as *S. aureus*, *E. faecium*, *E. coli*, *P. aeruginosa*, and *K.pneumoniae* [169]. The antibacterial activity of ZnONPs is attributed to the blockage of potassium ion channels on the microbial membrane that is performed by Zn^2+^ ions produced after their release in aqueous medium [170]. This leads to the destabilization of the membrane and microbial cell death. Moreover, the production of reactive oxygen species, binding to DNA and proteins, the blockage of DNA amplification, and the alteration of the expression of several genes have also been proposed as mechanisms of action of ZnONPs [170,171,172,173,174,175,176].

In a study by Vijayakumar et al., ZnONPs were synthesized from the methanolic leaf extract of *Glycosmis pentaphylla* [169]. Scanning electron microscopy revealed that most NPs were spherical in shape and had a diameter of 32–40 nm. The antimicrobial activity of these NPs against several Gram-positive and Gram-negative pathogens and fungi was studied at different concentrations. The maximum zone of inhibition was noted for the concentration of 100 μg/mL for all pathogens that were tested, namely, *Shigella dysenteriae*, *Bacillus cereus*, *Salmonella paratyphi*, *Candida albicans*, *A. niger*, *Staphylococcus aureus*, *Salmonella paratyphi*, and *Bacillus cereus* [169].

In another study, Reza Rajabi et al. used microwave-assisted extraction to allow the synthesis of ZnONPs from the aqueous extract of the *Suaeda aegyptiaca* plant [177]. Electron microscopy studies identified the NPs to be spherical with a size of 60 nm. The antimicrobial activity of ZnONPs was examined against *P. aeruginosa*, *E. coli*, *S. aureus*, and *B. subtilis*. For all these microorganisms, inhibitory and bactericidal activity was demonstrated, with minimum inhibitory concentrations of less than 0.5 mg/mL for *P. aeruginosa*, *S. aureus*, and *B. subtilis* and 1.56 mg/mL for *E. coli* [177].

Quantum dots are metallic and contain semiconductor materials as a core, like Zn or Cd. They have antimicrobial activity that is mainly attributed to the induction of membrane damage, the production of reactive oxygen species, damage to the microbial genetic material, and the inhibition of energy production [12,178]. In a relatively recent study, Courtney et al. synthesized 2.4 eV CdTe photoexcited quantum dots from NaHSe and NaHTe with CdCl_2_ and extensively described their properties [179]. Their size was in the range of 3 nm, and their antimicrobial activity against multidrug-resistant pathogens was evaluated. Quantum dots were able to kill many multidrug-resistant clinical isolates of bacteria, including methicillin-resistant *Staphylococcus aureus*, *Salmonella typhimurium*, carbapenem-resistant *Escherichia coli*, and extended-spectrumβ-lactamase (ESBL)-producing *Klebsiella pneumoniae*. Their bactericidal activity is independent of the material and appears to be controlled by the redox potentials of the charge carriers that are photogenerated and that selectively alter the cellular redox state [179].

In another study, Hao et al. developed a one-pot method for the preparation of positively charged carbon quantum dots that had a size of about 2.5 nm and showed potent antibacterial activity against many Gram-positive, Gram-negative, and drug-resistant bacteria. In a detailed investigation of the mechanism of their antimicrobial activity, the small-sized quantum dots strongly adhered to the bacterial cell membrane. Additionally, the entry of the quantum dots in the bacterial cells led to conformational changes in the DNA due to quantum dot adsorption, while reactive oxygen species were also produced intracellularly. Antimicrobial resistance against quantum dots was not detected. Moreover, quantum dots had significant antibacterial activity in a mixed infected wound rat animal model with *S. aureus* and *E. coli*, while the in vivo toxicity was minimal [180].

Organic NPs, such as polymeric micelles, liposomes, and other NPs, may carry either hydrophilic or hydrophobic antimicrobial agents [181]. Lipid NPs and liposomes are spherical vesicles consisting of phospholipid bilayers. These can fuse with the membranes of the target microorganisms, allowing for the delivery of the antimicrobial substance carried by the NPs in the pathogen [182]. Polymeric NPs, such as nanospheres, may also act as carriers of antimicrobial drugs [12]. For example, cellulose fibers reformed with AgNPs can act as potent antimicrobials against *S. aureus* and *E. coli* [183]. In another example, graphene quantum dots and silica nano-fabrications can lead to increased reactive oxygen species production after exposure to light by converting the energy of light to thermal energy, leading to potent bacterial killing [12]. Polymeric micelles include polystyrene, polylactic acid, poly(butyl methacrylate), poly(ethylene oxide), and poly(propylene oxide). These NPs generally have a hydrophobic inner core and a hydrophilic outer core. This allows them to be loaded with hydrophobic or hydrophilic antimicrobial drugs and to deliver them to the target microorganisms [184].

One example of an organic NP is encochleated amphotericin B. More specifically, due to several issues regarding the bioavailability of amphotericin B, encochleated amphotericin B has emerged as a potential solution towards the oral administration of this drug that had been traditionally used intravenously [138,185]. More specifically, amphotericin B is embedded within phosphatidylserine bilayers, thus creating a cochleate structure due to the action of calcium ions. This structure protects the drug from being digested in the gastrointestinal tract and allows for its absorption. Then, the cochleate structure is phagocytosed by human macrophages, and the bilayers pull open due to the difference in the calcium concentration, thus allowing for the intracellular delivery of the drug [138]. An in vivo study in a mouse model of cryptococcal meningoencephalitis showed that the oral encochleated amphotericin B with flucytosine had equal activity to parenteral amphotericin B with flucytosine and was superior to oral fluconazole without excess toxicity [185]. A phase-one study in HIV-positive survivors of cryptococcosis assessed the tolerability of the drug and proved that the oral encochleated amphotericin B was well tolerated when given in up to six divided daily doses without the occurrence of the toxicities that are commonly seen with the intravenous forms of amphotericin B [138]. Recently, a randomized clinical trial of encochleated amphotericin B (oral Lipid Nanocrystal Amphotericin B—MAT2203, Matinas Biopharma) showed promising results for the treatment of patients with cryptococcal meningitis with similar antifungal activity, survival, and less toxicity than the intravenous forms of amphotericin B [186,187].

Liposomal amphotericin B is one of the most widespread intravenous forms of amphotericin B and also consists of lipid-based NPs. With a size of about 80 nm, these NPs consist of liposomes carrying amphotericin B, thus reducing the likelihood of adverse events compared to other formulations [188]. The liposomes preferentially attach to the cell wall of the fungus and release the active amphotericin B molecule that is then transferred to the fungal cell membrane, where it can exert its activity, forming pores and leading to ion leakage and cell death [188]. Its efficacy and safety have been demonstrated in several studies of cryptococcal meningitis, candidiasis, visceral leishmaniasis, and invasive aspergillosis [188,189,190,191].

Nanozymes are NPs that have inherent enzymatic activity. Nanozymes were first reported in 2007, when ferromagnetic–oxide NPs were found to have inherent peroxidase-like activity and to be able to catalyze the peroxidase substrates 3,3,5,5-tetramethylbenzidine (TMB), di-azo-aminobenzene (DAB), and o-phenylenediamine (OPD) [94,192]. Nanozymes have lower cost and better stability compared to natural or other artificial enzymes, while their catalytic activity may be adjustable [193,194]. The most common enzymatic activity by nanozymes that could be used in the fight against infectious diseases is that of peroxidase, even though nanozymes with oxidase, haloperoxidase, or other enzymatic activities have also been implemented [192].

Qiu et al., in a recent study, developed a novel artificial enzyme possessing effective antibacterial activity and the potential for promoting wound healing [195]. They synthesized a hydrogel-based artificial enzyme consisting of copper and amino acids with an intrinsic peroxidase-like catalytic activity with a size of 50–70 nm that could effectively kill microorganisms in wounds and facilitate wound healing by increasing collagen deposition and angiogenesis [195]. Using in vitro studies, they proved that this nanozyme was effective against drug-resistant *S. aureus* and drug-resistant *E. coli*. Furthermore, they used local application of the nanozyme in a mouse model of an infectious wound and proved its efficacy towards wound healing [195].

In another example, Fang et al. synthesized a peroxidase mimic from nanodiamonds with a size of 2–10 nm to treat periodontal infections [196]. In vitro experiments revealed that the oxygenated nanodiamonds had potent antibacterial activity against *Fusobacterium nucleatum*, *Porphyromonas gingivalis*, and *S. sanguis*. In vivo experiments in a murine periodontal infection model showed that oral cavity flushing once a day with the combination of nanozyme and hydrogen peroxide could lead to accelerated wound healing around sites of periodontal infection in time [196]. Table 3 shows representative examples of the different NPs that have been used in the treatment of infectious diseases.

### 5.5. Nanoparticles and Biofilms

Biofilms are microbial communities formed on surfaces that are surrounded by a polymer matrix consisting of extracellular polymeric substances, such as extracellular DNA, proteins, and exopolysaccharides [197,198]. The bacteria in the biofilms may exist in two different forms, with bacteria alternating from one to the other during the formation and maturation of the biofilm [199]. In the planktonic form, the bacteria are metabolically active and are not attached to the surface of the biofilm, while the sessile bacteria are metabolically relatively inactive and are the ones that are attached to the surface of the biofilms and constitute their main mass [200]. This transition is associated with a great change in their gene expression profile, the reduction of their metabolic activity, and an associated significant increase in their antimicrobial resistance to common antimicrobials [201,202].

Bacterial biofilms could be a target for NPs since some of them appear to have potent activity against them. More specifically, SeNPs could retard the formation of biofilm by *P. aeruginosa* [203]. Moreover, in vitro tests with antimicrobials and biofilm tube ring formation studies show that ZnO NPs have significant activity against bacteria with significant AMR, such as *Staphylococcus* and *Klebsiella*, and they can also inhibit the formation of bacterial biofilm [204]. Additionally, large aggregated AgNPs can penetrate infected areas better, and they may also resemble longer retention in bacterial biofilms, thus leading to an inhibition of biofilm formation and even the effective elimination of the bacterial populations [12]. The aggregated AgNPs may also have a longer retention duration in the tissues, while exocytosis from the cells may be slower compared to that of small non-aggregated particles, allowing for a longer antimicrobial effect in the biofilms [12].

### 5.6. Combination of Nanoparticles with Antibiotics

**Figure 2:**
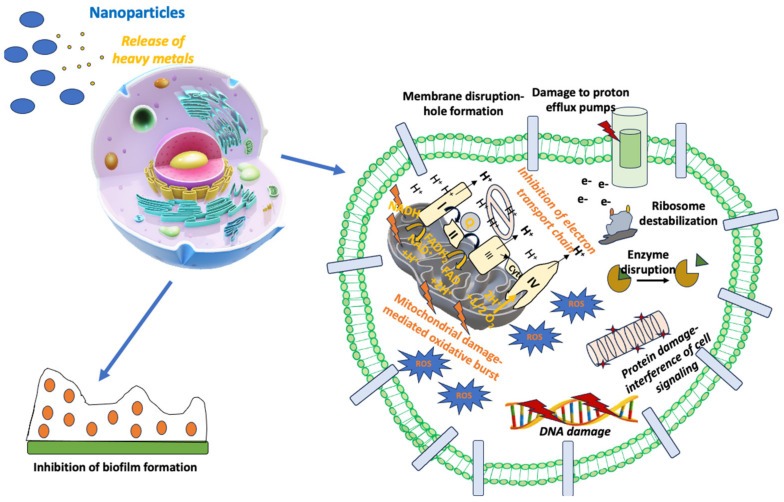
Mechanism of action of nanoparticles in the treatment of infectious diseases.

Combining NPs with already approved antibiotics may be a very promising option for the therapy of infectious diseases, especially for pathogens harboring significant AMR [205]. Several research groups have published reports studying different NPs in combination with antibiotics against many different bacteria since this effort can overcome AMR and reduce the toxic adverse effects of systemically administered antibiotics [206]. In this direction, Ag, Au, and ZnONPs conjugated with beta-lactams have shown strong bactericidal activity against *E. coli*, *S. aureus*, *A. baumannii*, *E. faecium*, and *P. aeruginosa* by their incorporation into the cell membrane and their interference with signal transduction pathways [206]. As a result, NPs seem to be effective in the fight against resistant Gram-positive and Gram-negative bacterial strains [206]. The different NPs based on Ag, Au, or Zn seem to potentiate the bactericidal effect of several antibiotics, like vancomycin, ceftazidime, clindamycin, ciprofloxacin, polymyxin B, and ampicillin, against bacterial strains with significant AMR [206]. Another mechanism explaining the synergistic effect of NPs with antibiotics is the enhancement of bacterial vulnerability to antibiotics. For example, NPs can allow antibiotics to penetrate into the bacterial cell membrane, thus facilitating its killing through the induction of microbial cell damage [207]. Hence, AgNPs have been combined with antibiotics like vancomycin, erythromycin, streptomycin, and chloramphenicol and have shown strong antimicrobial activity, achieving significant growth inhibition of Gram-positive and Gram-negative bacteria [208]. The bactericidal mechanism of AgNPs with the antibiotics mentioned above was dependent on the type of antibiotic used [208]. The most significant escalation of antibacterial action was shown by the streptomycin-conjugated AgNPs against *E. coli* [208]. Figure 2 summarizes the mechanisms of action of NPs in the treatment of infectious diseases.

### 5.7. Choosing the Right NP

Choosing the right antimicrobial substance for the treatment of a particular infectious disease, irrespective of whether it is a classic antibiotic, a bacteriophage, an antimicrobial peptide, an NP, or another substance, may be challenging. Several differences exist among the different NPs that were previously described herein. However, the pharmacological properties of the final drug may not be that easy to predict, and the possible adverse events may also differ from those that could be seen in a clinical trial in humans or in real-world data after the approval of the drug. Several such examples exist in cancer, as reviewed in a recent study by Mukherjee et al. [209]. Indeed, several issues regarding the translation of nanomedicines from bench to bedside exist and include issues regarding the production of the NP, its physicochemical characteristics, and issues regarding its pharmacokinetics, pharmacodynamics, and issues regarding long-term safety [210]. Thus, even though quantum dots may be more appropriate in the diagnosis of infectious diseases rather than in treatment, while the predominant NPs used for the treatment of infectious diseases that have been accepted for clinical use until now are lipid-based, the right choice of NP for the treatment of an infectious disease may require preclinical studies in animals and clinical studies in humans. For example, liposomal amphotericin B and encochleated amphotericin B are both lipid-based and seem to be effective in the treatment of fungal diseases [185,186,188,189,190,191]. However, encochleated amphotericin B seems to be stable for oral use and has adequate pharmacokinetics and pharmacodynamics, which is an obvious advantage compared to the other lipid-based forms of amphotericin B. Indeed, for two NPs with similar bioavailability, the one that can be administered orally would be preferable, since this would be associated with a reduced need for healthcare utilization, as well as fewer complications, such as those associated with maintaining an intravenous route of administration [211,212].

In the future, more NPs may be available for the treatment of infectious diseases and from many different NP categories. At that time, more parameters should be considered when choosing the appropriate NP for treatment. Factors such as bioavailability, cost, adverse events, required duration of treatment, and route of administration should be considered when deciding which NPs to use.

## 6. Nanotechnology in the Prevention of Infectious Diseases

### 6.1. Application of Nanotechnology in Vaccine Technology

Vaccines have changed the course of human history by significantly reducing the likelihood of specific transmissible diseases and even by eliminating some of them [213,214]. Even though much has been achieved with the vaccine technology that was previously available, further studies are being performed toward the optimization of vaccines by also implementing the latest advances in science [215,216].

NPs are being currently evaluated as a means to increase vaccine efficacy, increase antigen uptake, and produce a stronger immunologic response from the host against specific antigens [217,218]. NPs may have several advantages compared to other novel methods for vaccine development, such as nucleic acid vaccines, viral vector vaccines, and protein and synthetic peptide vaccines, such as the high stability, the low rate of serious adverse events, and the lack of requirement for an adjuvant [216]. They can also be designed to enhance specific cells of the immune system or intracellular compartments [218]. As in the case of their use as antimicrobial agents, their properties, such as their surface charge, hydrophobicity, and size, mainly define their function and, eventually, the efficacy of the immunological response produced by the antigen delivered by the NPs [216]. For example, the mechanism of the cellular uptake of the NPs and the antigen depends on the size of the NPs, which defines if phagocytosis, endocytosis, or micropinocytosis will be performed, thus affecting the immunogenicity [219,220].

The interaction between the NP and the antigen may involve three different mechanisms, namely antigen adsorption on the surface of the NP, covalent binding of the antigen to the NP (conjugation), and finally, antigen encapsulation in the NP. The type of interaction used depends on the nature of the antigen and the NP [216]. The most commonly used types of NPs in vaccine technology involve liposomes, polymeric NPs, emulsions, virus-like particles (VLPs), and immunostimulatory complexes (ISCOMs) [221].

VLPs are multi-protein nanostructures that are self-assembled by using viral structural proteins and closely resemble the formation of naturally occurring viruses. They can be formed in several different protein expression systems, such as in bacteria or mammalian cells. Since they do not have genetic material, they have no infective potential and are, thus, safe for administration in humans [216]. Their small size allows for efficient drainage in the lymph nodes [222,223], while, their stable structure allows for adequate uptake and antigen presentation by the antigen-presenting cells that lead to the activation of adaptive immunity [224].

Liposomes can carry antigens in their hydrophobic lipid bilayer or their hydrophilic core. Furthermore, some antigens can be attached to their outer layer by chemical cross-linking or by adsorption [225,226]. Additionally, liposomes can achieve more potent immunostimulatory properties by adding molecules like the monophosphoryl lipid A or lectin-binding mannose. This may increase delivery to antigen-presenting cells, like dendritic cells [227]. Finally, designing smaller or larger liposomes as methods of antigen delivery may affect the arm of the immunity that is mostly activated. For example, immunization with liposomes larger than 400 nm favored a Th1 immune response, while the use of smaller liposomes favored a Th2 response [228].

ISCOMs are cage particles with a size of about 40 nm that also self-assemble and can be used as delivery NPs for vaccines [216]. They are formed by the combination of a protein antigen, cholesterol, or phospholipids through the formation of hydrophobic interactions. They can entrap hydrophobic antigens and have been shown to effectively produce humoral and cellular immunity [229,230].

### 6.2. Limitations of Nanotechnology Applications in Vaccine Technology

NPs could increase the delivery of antigens and increase immune stimulation when applied in vaccine technology, as mentioned above. However, NPs can enter human organs and cells since they may easily cross membrane bilayers [231]. The clearance of NPs would depend on their size, but in general, it involves their phagocytosis from mononuclear phagocytes, kidney, and biliary excretion. Metallic NPs may negatively affect innate immunity through cytotoxicity and the interference of cytokine production, leading to the production of free radicals and reactive oxygen species, thus leading to cell death and, potentially, oncogenesis [231,232]. Due to their small size, their high surface-to-mass ratio, and the physicochemical properties of the NPs, they can increase phagocytosis of the antigen, thus increasing the immune response of the host against the antigen; however, they can generate adverse effects, such as necrosis or apoptosis, in other tissues. For example, intranasal immunization with NPs could be associated with the possibility of inducing lung injury through the induction of oxidative stress and the production of inflammatory cytokines and cytotoxic cellular responses [233]. Additionally, NPs can be aggregated, thus blocking the flow in the blood vessels of the host [233]. Finally, the possibility of germline alterations or carcinogenesis has been previously noted in animal models, in which VLPs could disperse widely in the body, thus ending up in the testes and ovaries of the host [234].

## 7. Challenges in the Use of Nanotechnology in the Treatment of Infectious Diseases

Applications of nanotechnology may have many advantages in the treatment of infectious diseases in the future; however, several shortcomings need to be addressed before these are widely implemented [235]. For example, further studies of the potential effects of NPs on human cells and tissues, issues regarding pharmacokinetics and pharmacodynamics, optimal dosing, potential short-term and long-term adverse events, and the optimal route of administration should be performed to allow for the safe and efficient use of NPs [154,236]. For example, the possibility for human toxicity by NP accumulation in the spleen, bone marrow, and lung exists after intravenous administration [237]. Furthermore, inhaled NPs may lead to accumulation and toxicity in the lung, liver, heart, spleen, liver, and brain [238]. This toxicity may be associated with the induction of oxidative stress and metabolic modifications, such as ketogenesis and beta-oxidation of lipids [238,239,240]. Thus, nanotechnology has led to very few FDA-approved medications until now. Finally, the cost-effectiveness of these therapies needs to be evaluated [235,241].

## 8. Conclusions

The increasing prevalence of AMR among Gram-positive and Gram-negative bacteria is associated with high morbidity and mortality. This, in combination with the relative reduction of the production of novel antimicrobials by the pharmaceutical industry, underlines the need for the identification of novel ways to fight infectious diseases with other means, beyond antimicrobials. To this end, nanotechnology emerges as a valuable tool both in the diagnosis and the treatment of infectious diseases. The ability of NPs to directly kill bacteria without being affected by AMR may allow for efficient treatment in cases with very few therapeutic options. However, the short-term and long-term safety, as well as the efficacy of NPs in the treatment of infectious diseases, should be confirmed in well-designed, randomized, and controlled trials in the future.

## Figures and Tables

**Table 1:** Comparison of different nanoparticle categories.

Nanostructure	Type of Structure	Properties and Advantages	Disadvantages	References
Dendrimers	Polymeric	Potent activity against cancer cells	Difficult to purify and produce in large quantities, potential toxicity	[57,58,59]
Drug conjugates	Polymeric	Provide controlled release, increase activity and tolerability of drugs	Extensive knowledge of polymer–receptor interaction required	[60,61]
Liposomes	Polymeric	Lipid bilayers incorporating drugs to enhance delivery, formation of macromolecular drugs with peptides, antibodies, or polymers, biodegradable	May form crystals during prolonged storage, high production cost, low solubility, short half-life	[13,38,43,51]
Micelles	Polymeric	Extremely small, amphiphilic polymers, increased aqueous solubility	Moderate loading capacity	[51,52,53]
Carbon nanotubes	Nonpolymeric	Increased drug stability and solubility, targeted drug delivery	Limited results from clinical studies, potential toxicity, variable pharmacokinetics	[36,62]
Metallic nanoparticles	Nonpolymeric	Controlled and targeted delivery, potential for contrast agents	Biocompatibility, not strong optical signal	[46,47]
Quantum dots	Nonpolymeric	Improved bioavailability and efficacy, high quality fluorescence	Toxicity of the core, relative instability, difficult to produce massively	[47,56,63]

**Table 2:** Examples of nanosensors that have been evaluated in the diagnosis of infectious diseases.

Nanoparticle	Pathogen	Detection Methodology	References
AgNPs	Dengue, Yellow Fever, and Ebola Viruses	Colorimetry	[92]
AgNPs	KSHV and *Bartonella*	Colorimetry	[93]
AgNPs	MERS-CoV, MTB, HPV	Colorimetry	[116]
AuNPs	*E. coli* and *S. pneumoniae*	Colorimetry	[87]
AuNPs	HBV	Colorimetry	[88]
AuNPs	SARS-CoV-2	Colorimetry	[117]
AuNPs	*E. coli* ATCC 8739	Fluorimetry	[118]
AuNPs	*E. coli* O157:H7	SERS	[119]
AuNPs	HIV	SERS	[109]
AuNPs, AgNPs	*Bartonella*, KSHV	Colorimetry	[93]
Silica nanoparticles, AuNPs	HIV	Electrochemiluminometry	[120]
Fe_3_O_4_@Au nanoparticles, AuNPs	*S. aureus*, *E. coli*	SERS	[121]
Carbon nanotube	*E.coli*, HPV	Electrochemiluminometry	[97,98]
Copper-based metal-organic framework (Cu-MOF) NPs	*S. aureus*	Colorimetry	[95]
AuNPs	HIV-1, KSHV and BA	SERS	[109,110]
CdSe@ZnS quantum dots	*E. coli*	Fluorimetry	[101]

Ag NPs: silver nanoparticles; Au NPs: gold nanoparticles; BA: bacillary angiomatosis; *E. coli*: *Escherichia coli*; KSHV: Kaposi’s sarcoma-associated herpesvirus; HBV: Hepatitis B virus; HIV: human immunodeficiency virus; HPV: Human Papillomavirus; *L. monocytogenes*: *Listeria monocytogenes*; MERS-Covid: Middle East respiratory syndrome coronavirus; MTB: Mycobacterium tuberculosis; *P. aeruginosa*: *Pseudomonas aeruginosa*; RSV: respiratory syncytial virus; *S. aureus*: *Staphylococcus aureus*; *S. typhimurium*: *Salmonella typhimurium*; SARS-CoV-2: severe acute respiratory syndrome coronavirus 2; SERS: surface-enhanced Raman spectroscopy; TGEV: Transmissible gastroenteritis virus; VSV: Vesicular stomatitis virus. The table is not exhaustive of all NPs that are available or under evaluation.

**Table 3:** Examples of nanoparticles that have been used in the treatment of infectious diseases.

Nanoparticle Category	Nanoparticle Type	Size	Zeta Potential	In Vitro Drug Release	In Vitro Results	In Vivo Results	References
Metallic NP	Polymer thin film loading CuNPs	3.2–5.3 nm, 80–530 nm for the nanocomposite films	NR	First-order process with an average kinetic constant of 0.014 ± 0.008/min	Clear biostatic activity on *Saccharomyces cerevisiae*, *Escherichia coli*, *Staphylococcus aureus*, and *Listeria monocytogenes* growth	NA	[155]
Metallic NP	Monodisperse spherical CuNPs	6–20 nm	NR	In vitro application after extracellular production from *Shewanella loihica* PV-4	100 µg/mL CuNPs inhibits 86% of *E. coli*	NA	[165]
Metallic NP	AgNPs (mostly spherical in shape)	6 nm and 18 nm	NR	In vitro application	200 mg/L had the highest inhibition effect on *Salmonella*	NA	[159]
Metallic NP	AuNPs (spherical)	80–120 nm	−41.4	In vitro application	Adequate antimicrobial activity against *S. typhi*, *P. aeruginosa*, *E. aerogenes*, *S. aureus*, *B. subtilis*, and *M. luteus*	NA	[167]
Metallic NP	AuNP (mostly spherical; also, triangular and hexagonal)	40–85 nm	NR	In vitro application	Adequate antimicrobial activity against *P. aeruginosa*, *K. oxytoca*, *E. faecalis*, *K. pneumoniae*, *V. cholerae*, *E. coli*, *S. typhi*, *S. paratyphii*, *V. parahaemolyticus*, and *P. vulgaris*	NA	[168]
Metallic NP	ZnONPs (mostly spherical in shape)	32–40 nm	NR	In vitro application	Maximum zone of inhibition at 100 μg/mL for *Shigella dysenteriae*, *Bacillus cereus*, *Salmonella paratyphi*, *Candida albicans*, *A. niger*, *Staphylococcus aureus*, *Salmonella paratyphi*, and *Bacillus cereus*	NA	[169]
Metallic NP	ZnONPs (spherical)	60 nm	NR	In vitro application	Inhibitory and bactericidal activity was demonstrated against *P. aeruginosa*, *E. coli*, *S. aureus*, and *B. subtilis*	NA	[177]
Quantum dots	2.4 eV CdTe photoexcited quantum dots	3 nm	NR	In vitro application	Quantum dots can kill MRSA, CR-*Escherichia coli*, and ESBL *Klebsiella pneumoniae* and *Salmonella typhimurium*	NA	[179]
Quantum dots	Positively charged carbon quantum dots	2.5 nm	−12.77 mV	In vitro application (in vitro); local application to the skin (in vivo)	Potent antibacterial effect on *S. aureus*, MRSA, *L. monocytogenes*, *E. faecalis*, *E. coli*, *S. marcescens*, *P. aeruginosa*, drug-resistant *E. coli*, and drug-resistant *P. aeruginosa*. Better antibacterial effect on Gram-positive bacteria	Faster healing and faster white blood cell recovery in a mixed infected wound rat animal model with *S. aureus* and *E. coli*, with minimal in vivo toxicity	[180]
Lipid-based	Encochleated amphotericin B (MAT2203, Matinas Biopharma)	NR	NR	Oral administration	NA	Potent anticryptococcal activity in mice and humans with favorable safety profile	[138,185,186]
Lipid based	Liposomal amphotericin B	80 nm	NR	Intravenous administration	NA	Potent activity in invasive aspergillosis, cryptococcal meningitis, and visceral leishmaniasis in humans	[188,189,190,191]
Nanozyme	Hydrogel-based artificial enzyme	50–70 nm	NR	Direct application (in vitro); local application to the skin (in vivo)	Activity against drug-resistant *S. aureus* and drug-resistant *E. coli*	Excellent wound healing properties in mice	[195]
Nanozyme	Oxygenated nanodiamonds	2–10 nm	−19–−36 mV	Direct application (in vitro); local application with oral cavity flushing (in vivo)	Potent activity against *Fusobacterium nucleatum*, *Porphyromonas gingivalis*, and *S. sanguis*	Acceleration of wound healing after periodontal infection	[196]

ESBL: extended-spectrum β-lactamase; MRSA: methicillin-resistant Staphylococcus aureus; NA: not applicable; NP: nanoparticle; NR: not reported; the table is not exhaustive of all NPs that are available or under evaluation.
